# When music tells a story: children’s language and socioemotional development through music-supported narrative learning

**DOI:** 10.3389/fpsyg.2026.1956337

**Published:** 2026-09-17

**Authors:** Rong Ma, Chuan Chen

**Affiliations:** 1 College of Chinese & ASEAN Arts School of Music and Dance, Chengdu University, Chengdu, China; 2 College of Music and Dance, Sichuan Technology and Business University, Chengdu, China

**Keywords:** childhood, language–narrative development, music education, music-supported narrative learning, narrative competence, socioemotional development, systematic review

## Abstract

**Background:**

The WHO Regional Office for Europe has increasingly recognized the potential value of arts and music participation for children’s health, well-being, and development. However, how music supports children’s learning when integrated with narrative activities remains insufficiently understood.

**Objective:**

This review examined the practice structures, language–narrative and socioemotional outcomes, and learner- and design-related conditions associated with music-supported narrative learning for children aged 3–12 years.

**Methods:**

Following PRISMA 2020, Scopus, Web of Science, and ERIC were searched for English-language original empirical studies published from January 2016 to August 2026. Nineteen studies were included. Inductive thematic synthesis, outcome-oriented narrative synthesis, and cross-study explanatory synthesis were used. Quantitative and qualitative evidence were synthesized separately before integration, and cross-study relationships were interpreted as patterns of co-occurrence.

**Results:**

Practices included auditory enhancement of predefined narratives, sung or rhythmized text, embodied and multimodal activities, and generative music–narrative creation. Language–narrative findings were heterogeneous. More favorable outcomes were reported in some studies when music was functionally aligned with target language or narrative content, but this pattern was inconsistent. Socioemotional evidence more often concerned expressive, participatory, and relational affordances, especially in generative and collaborative practices.

**Conclusion:**

This review integrates language–narrative and socioemotional evidence within a common framework and identifies two emerging patterns—information organization and expression–relationship—shifting attention from whether music works to which musical functions and participation structures relate to particular outcomes, although these patterns require further validation.

## 1 Introduction

A large-scale scoping review commissioned by the WHO Regional Office for Europe synthesized broad evidence on the relationships between arts engagement, health, and well-being, including research involving music participation among children and adolescents (Fancourt and Finn, 2019). More recent meta-analytic evidence on children’s music education suggests that music training may also be associated with executive-function development, although outcomes vary across developmental stages, cognitive domains, and instructional structures (Wang et al., 2026). Together, these findings provide a broad rationale for expanding children’s access to structured music education, but they do not indicate which specific forms of musical instruction are appropriate for particular developmental goals. In particular, previous reviews have not systematically examined how the informational function of music, children’s participation structure, and narrative-content context jointly relate to language–narrative and socioemotional outcomes when music is integrated with narrative learning.

Narrative learning enables children to organize characters, events, causal relations, and emotional experiences into coherent structures of meaning and therefore provides an important foundation for language comprehension, narrative expression, and the construction of social experience (Bruner, 1990; Pico et al., 2021). Music, in turn, offers distinctive temporal, rhythmic, and emotional resources for narrative learning. Rhythm, melody, and repetition can support children’s processing of phonological, semantic, and syntactic information (Neves et al., 2022; Pino et al., 2023), while musical activities may also facilitate emotional expression, engagement, and social connection (Blasco-Magraner et al., 2021). Music-supported narrative learning may therefore be associated with both children’s language–narrative and socioemotional development.

However, the outcomes reported in the existing literature are not uniformly positive. Music-supported narrative learning is not a single instructional model. Rather, it encompasses a range of practices, including auditory enhancement in digital storybooks, sung book-sharing, rhythmized recitation, songwriting, and embodied or multimodal narrative activities. These practices differ substantially in their instructional structure, forms of child participation, and contextual organization. Recent research in music education has likewise suggested that developmental and non-cognitive outcomes may depend on configurations of instructional and contextual conditions rather than on any single educational component in isolation (Choe et al., 2025; Dweck, 2024; Li and Bus, 2023; Liu et al., 2022; Shan et al., 2025). It is therefore necessary to systematically identify the structural and contextual differences among these practices and, on that basis, synthesize the language–narrative and socioemotional outcomes associated with them. Organizing specific instructional formats into comparable design features may provide a more systematic basis for interpreting heterogeneous findings and for generating testable hypotheses for curriculum design and implementation.

Learner characteristics may also influence the outcomes of music-supported narrative learning. For example, the simultaneous presentation of auditory and visual enhancements may not be more beneficial for younger children (Li and Bus, 2023). Reading gains among immigrant children participating in structured singing activities may be related to length of residence, home language, and prior literacy experience (Choe et al., 2025). The effects of sung book-sharing on parent–child attention may also differ according to children’s neurodevelopmental characteristics and language ability (Liu et al., 2022). Together, these studies suggest that reported outcomes may vary across combinations of practice conditions and learner characteristics. However, because these characteristics and practice conditions are often structurally clustered across studies, their independent relationships with outcomes remain unclear.

In this review, music-supported narrative learning refers to structured activities in which music or musical elements are functionally integrated with an identifiable narrative process. Consistent with established accounts of narrative development and intervention, such processes may involve story comprehension, narrative organization, retelling, generation, enactment, or the reconstruction of experience (Bruner, 1990; Pico et al., 2021). Personal or emotional expression alone was not considered narrative learning unless it was organized around identifiable characters, events, experiences, or relationships. Music-supported narrative learning nevertheless encompasses considerable variation in how music is organized within narrative activity, how children participate in meaning-making, and what kinds of experiences provide the narrative content. Accordingly, the review examines three interrelated dimensions: instructional components, participation structures, and narrative-content contexts. Instructional components refer to the functional organization of music and other activity elements within the narrative task; participation structures refer to children’s roles, forms of interaction, and degree of control over content, consistent with sociocultural perspectives emphasizing guided participation and participation in socially organized activity (Rogoff, 1990); and narrative-content contexts refer to the source and experiential domain of the narrative material. Together, these dimensions provide a common analytical framework for examining how activity organization, child participation, and narrative content vary across music-supported narrative learning practices. The review addresses the following research questions:

*RQ1*: What instructional components, participation structures, and narrative-content contexts characterize music-supported narrative learning practices?

*RQ2*: Which language–narrative and socioemotional outcomes are associated with music-supported narrative learning practices for children?

*RQ3*: Which practice features and learner characteristics are associated with differences in the outcomes reported across music-supported narrative learning practices?

## 2 Theoretical framework

### 2.1 Narrative development theory

Narrative development theory conceptualizes narrative as the process through which children organize characters, events, goals, actions, and outcomes into coherent structures of meaning. This process involves linguistic organization, memory integration, causal reasoning, and social cognition (Bruner, 1990; Stein and Glenn, 1979). Children’s narrative competence is commonly understood as comprising two interrelated levels. Macrostructure concerns the overall organization of story setting, character goals, event progression, and outcomes, whereas microstructure concerns linguistic resources such as lexical diversity, syntactic complexity, reference, temporality, and causal cohesion. Recent research continues to use this distinction and indicates that both levels are influenced by age, language experience, and task type (Lindgren, 2023; Lipner et al., 2024).

Narrative competence can be developed through explicit instructional support. Systematic reviews and meta-analyses indicate that narrative interventions focusing on story structure, causal relations, and expressive language strategies can improve children’s narrative comprehension and production. Similarly, the benefits of interactive reading appear to derive primarily from questioning, retelling, feedback, and structured scaffolding rather than from simple exposure to story materials (Pico et al., 2021; Xing et al., 2025).

Narrative development also involves understanding characters’ goals, emotions, and perspectives. Children must connect characters’ actions and psychological states with event outcomes in order to construct a more complete representation of a story. Narrative activities therefore involve not only language and story organization but also emotional understanding and perspective-taking. Music with different emotional characteristics can influence children’s interpretations of story situations (Ziv and Goshen, 2006), suggesting that music may contribute to the construction of narrative meaning.

### 2.2 Sociocultural learning theory

Sociocultural learning theory proposes that children’s higher psychological functions develop gradually through interaction with adults, peers, and cultural tools. Language, symbols, and artistic forms operate as cultural mediational tools through which meanings and strategies embedded in shared activity are progressively transformed into individual capabilities (Vygotsky and Cole, 1978). Teacher scaffolding and guided participation are central to this process. Through modelling, questioning, feedback, and the gradual transfer of responsibility, adults support children’s participation in meaning-making during shared reading, discussion, and collaborative creation (Rogoff, 1990).

From this perspective, music is not merely an additional sound layer attached to a story; it can function as a cultural tool through which narrative participation and shared meaning-making are organized. In music-supported narrative learning practices, language, singing, rhythm, movement, and other semiotic resources can be coordinated within socially situated activity, providing children with different opportunities for expression, interaction, and collaborative participation (Rowe, 2010). Musical activities may also be associated with emotional expression, regulation, and prosocial development, although these relationships may vary according to activity format, participation structure, and educational context (Blasco-Magraner et al., 2021). This perspective therefore highlights the importance of examining not only what musical resources are present, but also how they are organized within children’s participation and interaction.

### 2.3 Music-specific processes in narrative learning

Music may contribute to narrative learning through properties that are particularly salient in musical activity. Rhythmic and prosodic timing can organize linguistic material and support the processing of temporal structure (Destro Pastizzaro et al., 2026; Gordon et al., 2015), while repeated melodic organization may provide organizational and retrieval cues for verbal material (Wallace, 1994). More recent systematic evidence also suggests that song-based instruction may support multiple domains of language learning, although methodological limitations constrain strong causal conclusions (Hamilton et al., 2024). Music can convey affective information through acoustic features such as tempo, intensity, pitch, and melodic organization, thereby shaping emotional interpretation (Juslin, 2025). Moreover, musical rhythm and joint music-making are closely linked with interpersonal coordination, embodied synchronization, and shared participation (Abalde et al., 2024; Cheng et al., 2024; Tzanaki et al., 2025). In more generative practices, singing, songwriting, and musical enactment can therefore serve as structured resources through which children organize and express narrative, personal, or social meaning.

These processes are not assumed to be uniformly beneficial. Cognitive load theory provides an important boundary perspective: when additional auditory information does not contribute to the learning task, it may compete with verbal information for limited processing resources (Sweller, 1988). Research on multimedia learning has similarly shown that irrelevant music and sounds can interfere with learning (Moreno and Mayer, 2000). Self-determination theory offers a complementary perspective on participation, proposing that autonomy, competence, and relatedness constitute basic psychological needs relevant to motivation and well-being (Ryan and Deci, 2000). It therefore provides a basis for interpreting why receptive, collaborative, and child-directed music-supported narrative learning practices may provide different opportunities for agency, engagement, and belonging.

Taken together, narrative development theory and sociocultural learning theory provide the main explanatory foundation for this review, supporting the interpretation of developmental outcomes in relation to musical functions, children’s participation, and interactional support. Attention to the temporal, affective, embodied, and participatory processes of music further helps explain why different forms of musical practice may be associated with different outcomes.

## 3 Methods

### 3.1 Literature search strategy

The searches were conducted using Scopus, Web of Science Core Collection, and ERIC. The literature search and study-selection process followed the PRISMA 2020 guidelines. The initial database search was conducted on July 24, 2026, using Scopus, Web of Science Core Collection, and ERIC, and identified 1,892 records. Following revision, a final expanded and supplementary search was conducted through August 22, 2026. This update yielded 3,129 records and identified three additional eligible studies, increasing the final sample from 16 to 19 studies.

The search strategy was structured around four concept blocks: (1) music, (2) educational or intervention context, (3) narrative practice, and (4) language–narrative or socioemotional outcomes. Terms within each concept block were combined using “OR,” whereas the four concept blocks were combined using “AND.” Search strings were adapted to the field codes and syntax of each database. Language, publication-period, and publication-type restrictions were applied uniformly after record export and before deduplication. The complete database-specific search strings, search fields, limits, and final search date are provided in Supplementary File 1 (Table 1).

The review protocol was not prospectively registered in PROSPERO, OSF, or any other public repository. The review was reported in accordance with PRISMA 2020, and the completed PRISMA 2020 checklist is provided in Supplementary File 2.

### 3.2 Study screening and eligibility assessment

Of the 3,129 records retrieved in the updated search, 1,719 were from Scopus, 1,040 from Web of Science, and 370 from ERIC. After record export, 1,850 records were removed because they did not meet the predefined publication-type, publication-period, or language restrictions, including ineligible publication types, publications before 2016, and non-English publications. The remaining 1,279 records were then deduplicated. After 276 duplicate records were removed, 1,003 unique records remained for title and abstract screening (Figure 1).

**Figure 1:**
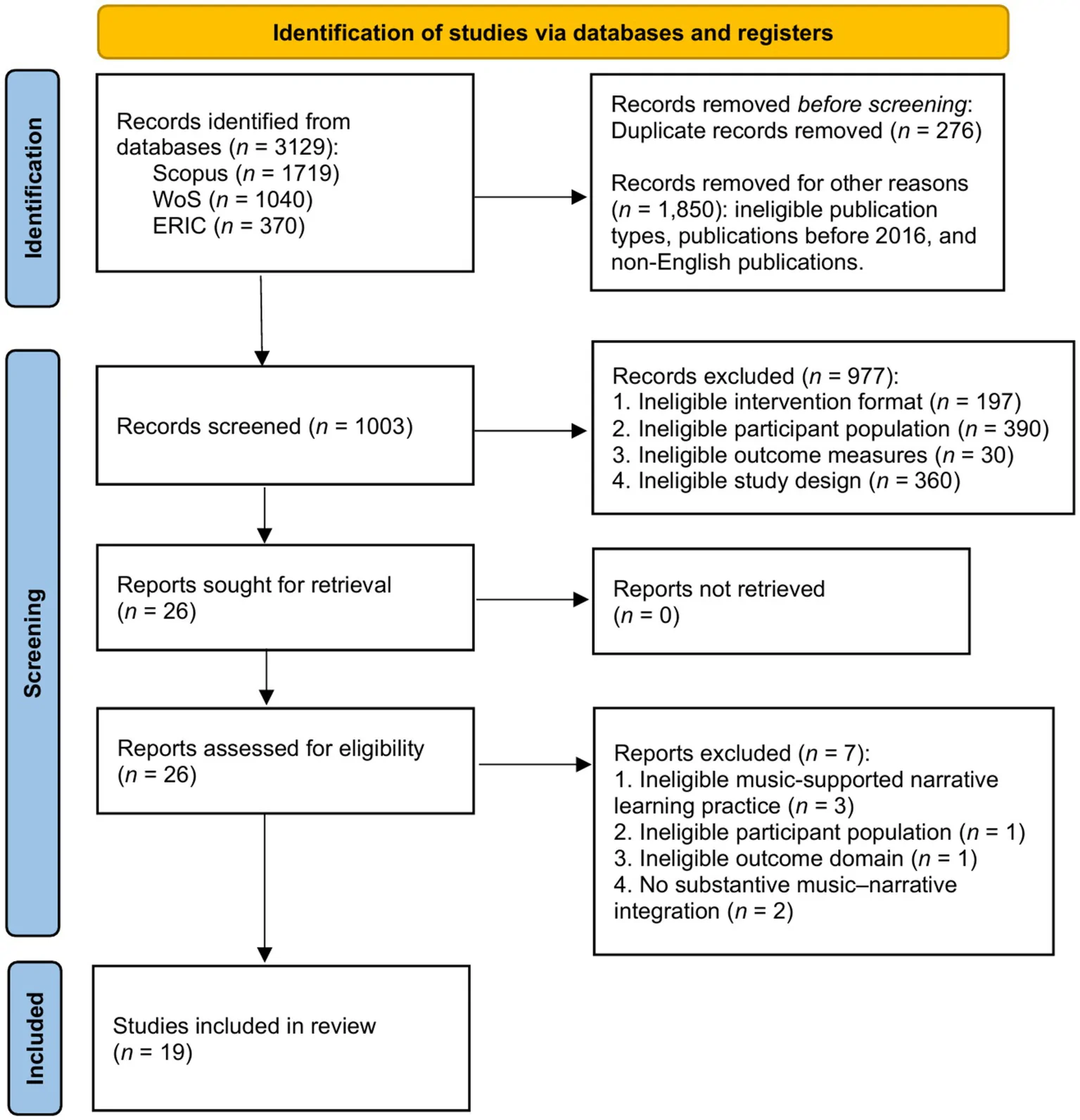
PRISMA flow diagram of the study selection process.

For eligibility assessment, the definition of music-supported narrative learning was operationalized using two necessary criteria: (1) the activity contained an identifiable narrative process, and (2) music or musical elements performed an identifiable function within that process rather than merely occurring alongside it. These criteria were used to determine whether a study contained an eligible focal activity and were not based on participant diagnosis or institutional setting.

In the first stage, titles and abstracts were screened to determine whether the participants belonged to the target child population, whether the activity involved a substantive integration of music with story or narrative, whether at least one language–narrative or socioemotional outcome was reported, and whether the publication presented original empirical research. Records with insufficient abstract information but no clear basis for exclusion were retained for full-text assessment. A total of 977 records were excluded at this stage, leaving 26 reports for full-text eligibility assessment.

In the second stage, the full texts were examined to determine whether participants were primarily children aged 3–12 years; whether music formed a core part of storytelling, narrative generation, lyric creation, or dramatic expression rather than functioning only as an unrelated background stimulus; whether the setting and purpose of the activity involved learning, communication, experiential expression, or social participation; whether the study used an eligible empirical design; and whether it reported at least one language–narrative or socioemotional outcome. Full-text reports were also required to provide sufficient information about procedures, measures, and findings to support eligibility assessment, methodological appraisal, and evidence synthesis. Seven reports were excluded at this stage, resulting in a final sample of 19 included studies (see Table 1).

**Table 1:** Study inclusion and exclusion criteria.

Screening dimension	Inclusion criteria	Exclusion criteria
Participants	Participants were primarily children aged 3–12 years, covering preschool and primary-school populations. Mixed-age studies were included only when data for children within the target age range could be extracted separately or when children aged 3–12 years constituted the primary sample.	Participants were not primarily children aged 3–12 years, and data for the target age group could not be extracted separately.
Participant characteristics and intervention purpose	Typically developing children and children with neurodevelopmental differences, clinical diagnoses, emotional or behavioral difficulties, bereavement, trauma, migration, displacement, or other psychosocial risk experiences were eligible. Participant diagnosis or psychosocial status was not itself a basis for inclusion or exclusion; eligibility was determined by the structure and function of the focal activity.	Studies were excluded when the focal activity was designed solely for clinical symptom management, physiological regulation, relaxation, or medical treatment and did not contain an identifiable narrative process functionally supported by music.
Practice or intervention format	The activity contained an identifiable narrative process, such as story comprehension, organization, retelling, generation, enactment, or the narrative reconstruction of personal or social experience, and music or musical elements had a substantive functional relationship with that process. This relationship could involve organizing narrative or linguistic material, supporting narrative participation or enactment, expressing or reinforcing characters and emotions, or contributing to narrative meaning-making. Eligible practices could therefore include musical storytelling, story songs, sung or rhythmized narrative texts, musical picture books, music-supported story retelling or generation, lyric or songwriting, musical drama or role-play, and multimodal narrative creation involving sound, rhythm, melody, or song.	Studies were excluded when no identifiable narrative process was present, or when music merely co-occurred with the narrative activity without an identifiable narrative function. This included unrelated background music, general singing or instrumental training without a narrative component, and music used solely for relaxation or emotional soothing.
Implementation setting	Eligible settings included mainstream schools, early childhood and inclusive education, family- or community-based learning activities, and supportive or therapeutic programmes. Setting itself did not determine eligibility; studies conducted in supportive or therapeutic contexts were included when the focal activity met the same music–narrative criteria applied to educational settings.	No setting was excluded solely because it was clinical or therapeutic. Studies were excluded when the focal activity failed to meet the music–narrative criteria, including programmes limited to symptom treatment, physiological rehabilitation, or medical care without an identifiable music-supported narrative process.
language–narrative outcomes	At least one outcome related to vocabulary, oral language or expression, literacy, reading fluency, story comprehension or recall, narrative competence, narrative production, communication within narrative activity, or multimodal narrative expression.	The study assessed only musical ability, general memory, motor development, physiological indicators, or academic outcomes unrelated to language–narrative development.
Socioemotional outcomes	At least one outcome related to emotional expression or regulation, self-evaluation, self-esteem, self-concept, self-efficacy, perceived control, psychological safety, prosocial behavior, social competence, peer or family relationships, belonging, resilience, identity, agency, or overall well-being.	The study reported neither an eligible language–narrative outcome nor an eligible socioemotional outcome.
Study design	Original empirical research, including randomized or non-randomized intervention studies, single-group pretest–posttest studies, observational studies, qualitative studies, mixed-methods studies, pilot studies, and case studies providing sufficient information on research procedures and findings.	Reviews, theoretical or conceptual papers, commentaries, books or book chapters, and conference abstracts or posters lacking complete empirical methods and findings.
Publication characteristics	Full-text empirical studies published in English between January 2016 and August 2026.	Studies published before 2016, non-English publications, or publications for which the full text was unavailable or could not be adequately assessed.

Eligible studies were original empirical studies in which children were the primary participants, the focal activity met the two operational criteria described above, and at least one language–narrative or socioemotional outcome was reported. Studies could be conducted in mainstream schools, early childhood or inclusive education, family- or community-based settings, and supportive or therapeutic programmes. Clinical diagnosis, neurodevelopmental difference, or psychosocial risk was not itself a basis for exclusion; rather, eligibility depended on whether music had a substantive functional relationship with an identifiable narrative process. This relationship could involve organizing narrative or linguistic material, supporting narrative participation or enactment, expressing characters or emotions, or contributing to narrative meaning-making. Accordingly, supportive or therapeutic studies were eligible when these conditions were met, whereas activities using music solely for relaxation, emotional soothing, physiological regulation, symptom management, or medical treatment without an identifiable narrative process were excluded. Likewise, music that merely co-occurred with a narrative activity as unrelated background accompaniment was not classified as music-supported narrative learning. If such a condition appeared as a comparator within an otherwise eligible multi-condition study, it was treated only as comparative evidence. The full eligibility criteria are presented in Table 2.

**Table 2:** Practice structures and types of music-supported narrative learning.

Analytical dimension	Type	Specific forms and classification boundaries	Corresponding studies
Core instructional components	Auditory enhancement of predefined narratives	Background music, environmental sounds, or auditory cues corresponding to plot events or characters’ emotions were added to existing picture books, stories, or animations. The narrative and musical materials were mainly prepared in advance by researchers or instructional designers, and children did not participate in creating the musical content.	S1, S4
	Sung and rhythmized presentation of text	Existing words, sentences, reading passages, or picture-book content were transformed into singing, rhythmic chanting, or melodic expression. The central feature was the use of rhythm and melody to process, repeat, or participate in existing texts rather than to generate new narrative content.	S7, S8, S14, S17
	Embodied and multimodal narrative activities	Music, rhythm, or singing was combined with movement, drama, drawing, clay work, puppetry, shadow play, yoga, digital media, or bodily imitation. Children mainly responded to or represented stories through bodily, visual, auditory, or digital modes.	S5, S6, S9, S11, S12, S19
	Generative music–narrative creation	Children created, revised, or jointly produced lyrics, songs, sounds, digital stories, or recorded products. Activities included collaborative songwriting, original lyrics and melodies, name songs, responsive song modification, musical improvisation, multitrack recording, and digital picture-book production.	S2, S3, S7, S9, S10, S13, S15, S16, S18, S19
Participation structure	Receptive participation	Children mainly watched or listened to predefined stories, music, songs, or digital picture books without changing the existing content.	S1, S4, S12, S14
	Responsive participation	Children responded to content predefined by adults or instructional materials through singing along, rhythmic chanting, answering questions, discussion, movement imitation, dramatic performance, drawing, or other guided multimodal activities. However, the topic and main task remained determined by adults or instructional materials.	S5, S6, S7, S8, S9, S10, S11, S12, S13, S14, S17
	Collaborative generative participation	Children jointly created lyrics, songs, stories, drawings, or digital products with peers. These activities required them to share ideas, negotiate content, and produce a common outcome.	S2, S3, S5, S9, S12, S15, S18, S19
	Child-led generative participation	Children had meaningful decision-making authority over narrative topics, personal experiences, lyrics, emotions, sounds, visual content, song revisions, or the direction of product development.	S2, S3, S5, S6, S9, S10, S13, S15, S16, S18, S19
Narrative-content context	Fictional and curriculum-based content	Narratives were drawn from fairy tales, published picture books, electronic stories, reading materials, target vocabulary, second-language texts, or curriculum-based educational stories.	S1, S4, S6, S7, S8, S11, S14, S17, S18
	Personal life and socioemotional experience	Narratives were based on children’s family life, school experiences, friendships, development, everyday emotions, self-esteem, safety, and general social relationships.	S3, S9, S11, S12
	Identity, family, and cultural experience	Narratives focused on names, self-identity, family history, local legends, traditional music, community culture, or cultural transmission.	S5, S8, S13, S15, S19
	Adversity, migration, and recovery experience	Narratives addressed bereavement, abuse, terrorism, displacement, immigrant or refugee adjustment, severe emotional difficulties, and relational repair.	S2, S5, S7, S9, S10, S16

To enhance the reliability of study screening, data extraction, coding, and quality appraisal, the review employed a combination of independent assessment, cross-checking, and consensus procedures across stages. The two reviewers first jointly clarified the eligibility criteria and conducted pilot screening to refine the operational screening boundaries. During title and abstract screening, Reviewer 1 screened all records, while Reviewer 2 independently screened a 20% subset selected using a computer-generated random sequence; inter-reviewer agreement was strong (Cohen’s *κ* = 0.87). All 26 full-text reports were subsequently assessed independently by both reviewers, with a pre-consensus agreement rate of 92.3%. Descriptive data were extracted by Reviewer 1 and fully checked by Reviewer 2, whereas interpretive coding of practice characteristics, outcome-category coding, and Mixed Methods Appraisal Tool (MMAT) appraisal of all included studies were conducted independently by both reviewers. Any disagreement in eligibility, coding, or MMAT item-level judgments was resolved by returning to the full text and relevant source evidence, discussing the applicable coding or appraisal criterion, and reaching consensus. Detailed reviewer roles and reliability procedures at each stage are presented in Supplementary File 1 (Table 2).

### 3.3 Quality appraisal

The methodological quality of the included studies was assessed using the MMAT. The MMAT is suitable for systematic reviews that include qualitative studies, randomized controlled trials, non-randomized studies, and mixed-methods research because it provides design-specific appraisal criteria (Hong et al., 2018).

Each included study was appraised against the five MMAT criteria corresponding to its study design, with each criterion rated as “Yes,” “No,” or “Cannot tell.” The complete criterion-level MMAT appraisal for all included studies is presented in Supplementary File 1 (Table 3).

The appraisal indicated substantial methodological heterogeneity across the included studies. Many individual studies met several or most of the MMAT criteria applicable to their design, but the strength of the evidence varied across studies and methodological domains. Common limitations included small sample sizes; single-case studies; retrospective interviews; single-group pretest–posttest designs; insufficient control of potential confounding variables in some non-randomized studies; incomplete integration of quantitative and qualitative findings in some mixed-methods studies; and limited reporting of assessor blinding or baseline comparability in some experimental studies. At the same time, some studies showed clear alignment among the research questions, data-collection procedures, and analytic approaches, and employed comparison groups, repeated measurements, multiple data sources, or longer-term observation.

Overall, methodological quality varied across the included studies, with strengths and limitations differing across individual MMAT criteria and study designs.

### 3.4 Data extraction and analysis

First, a standardized form was used to extract and organize the basic information from each study and the information related to the research questions. This information included the study title, author(s) and publication year, country or region, learning stage, instructional components, participation structure, narrative-content context, language–narrative outcomes, socioemotional outcomes, practice features, and learner characteristics. Descriptive information about the instructional process, forms of participation, and study outcomes was summarized and coded in a consistent way while retaining the original meaning as closely as possible. Different synthesis methods were then used for the three research questions. Supplementary File 1 (Table 4) presents the main study characteristics and analytical codes after the information was organized.

For RQ1, an inductive thematic synthesis was conducted. Information was first extracted on the narrative materials, forms of music and how they were used, children’s activities, adult and peer participation, activity organization, and implementation context. The extracted information was then openly coded. Codes with similar meanings were combined through constant comparison, and broader categories of practice were developed while remaining close to the descriptions in the original studies (Thomas and Harden, 2008).

For RQ2, an outcome-oriented narrative synthesis was conducted. Outcome measures, measurement approaches, main findings, and the direction and magnitude of reported findings were first extracted, and similar outcomes were grouped into the two broad domains of language–narrative development and socioemotional development. Because quantitative and qualitative studies provided different forms of evidence, the two evidence types were initially synthesized separately. For quantitative studies, interpretation focused primarily on the direction and actual magnitude of between-group differences or pre–post changes, together with effect sizes, confidence intervals, and statistical significance where these were reported. Statistical significance was not treated as the sole indicator of the direction or strength of evidence. Sample size, study design, and the precision of estimates were also considered when interpreting quantitative findings, while methodological limitations identified through the MMAT appraisal were used to inform the boundaries of interpretation. For qualitative studies, findings were synthesized from participant accounts, behavioral observations, researcher-reported themes, and other qualitative evidence provided in the original studies. In particular, observations concerning participation, expression, agency, interaction, or meaning-making were distinguished from measured developmental outcomes. Such process-level observations were not treated as evidence of developmental change unless corresponding changes in language–narrative or socioemotional functioning were directly measured or clearly documented.

After the quantitative and qualitative evidence had been synthesized separately, the two evidence streams were integrated at the level of common outcome domains and recurring cross-study patterns. Quantitative evidence was used primarily to summarize the direction and magnitude of measured outcomes, whereas qualitative evidence was used to elaborate children’s experiences, participation processes, and practice contexts, thereby extending the interpretation of observed patterns. Convergence, complementarity, and divergence between the two forms of evidence were then examined across outcome domains.

For RQ3, cross-study relationship exploration and explanatory synthesis were conducted. Based on the analyses for RQ1 and RQ2, a matrix was developed to connect practice features, learner characteristics, implementation contexts, and outcome patterns. Cross-study comparisons were then used to identify recurring practice characteristic–outcome combinations (Miles et al., 2020; Popay et al., 2006). The analysis also considered whether these patterns were supported primarily by quantitative evidence, qualitative evidence, or both, while taking study design and implementation context into account. Because these relationships were identified mainly through comparisons across heterogeneous studies, they were interpreted as recurring patterns of co-occurrence rather than as causal or mechanistic relationships. These patterns were therefore used to generate emerging explanatory patterns and hypotheses for future direct testing.

## 4 Results

### 4.1 Practice structures and types

Across the included studies, music-supported narrative learning was not implemented as a fixed instructional model. Instead, it involved different combinations of narrative materials, musical media, children’s participatory activities, and multimodal expressive tasks. The practices differed substantially in the instructional function of music, the degree of children’s participation and control over content, and the experiential sources of the narrative content. Overall, they formed a continuum ranging from receptive engagement with predefined content to the collaborative or child-led creation of music–narrative products (see Table 2; Figure 2). Figure 2 should be read as an evidence map showing how the identified practice structures are distributed across the principal outcome domains. More densely represented cells indicate that a particular practice–outcome combination appeared more frequently across the included studies, rather than that the corresponding practice produced a stronger effect. Because the practice codes were not mutually exclusive, individual studies may contribute to more than one cell.

**Figure 2:**
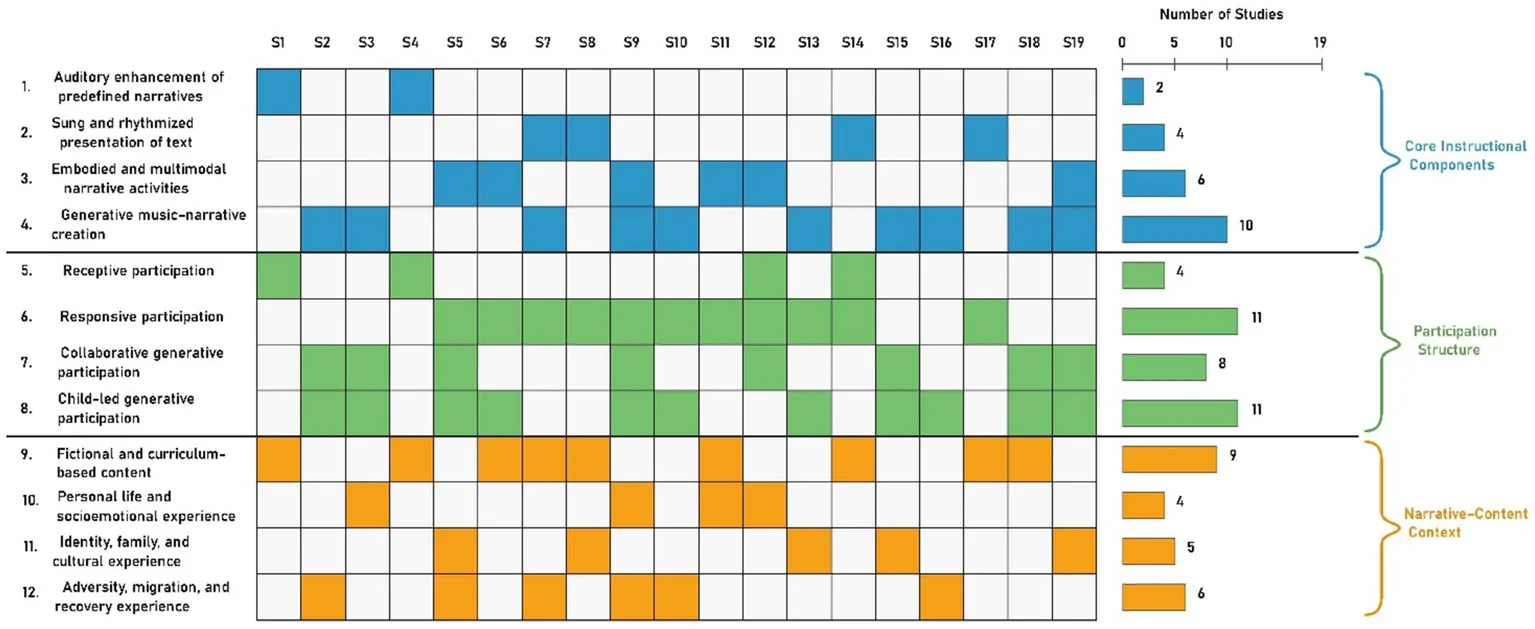
Evidence heatmap of music-supported narrative practices.

In terms of core instructional components, generative music–narrative creation was the most common, appearing in 10 studies. Embodied and multimodal narrative activities appeared in six studies, while auditory enhancement of predefined narratives and sung or rhythmized presentation of text appeared in two and four studies, respectively. The instructional components were not mutually exclusive. For example, some studies combined the rhythmization of existing texts, children’s creative production, and multimodal expression.

Regarding participation structure, responsive participation and child-led generative participation each appeared in 11 studies, collaborative generative participation appeared in eight studies, and receptive participation appeared in four studies. The overlapping codes indicated that children’s participation was not generally confined to a single level but could range from adult-guided responses to peer collaboration or child-led creation.

With respect to narrative-content context, fictional and curriculum-based content was the most common category, appearing in nine studies. Adversity, migration, and recovery experiences appeared in six studies; personal life and socioemotional experiences appeared in four studies; and identity, family, and cultural experiences appeared in five studies. Some studies spanned multiple narrative-content contexts, incorporating curriculum content, personal life, cultural identity, or difficult experiences within the same music-supported narrative learning practice.

Beyond these practice structures, the included studies also differed substantially in their implementation contexts and professional practice backgrounds. To account for this contextual heterogeneity, practice context was additionally coded as a supplementary analytic dimension, including mainstream or early-childhood education, inclusive education, family/community settings, and supportive or therapeutic programmes. These contexts differed in activity goals, practitioner roles, programme intensity, learner characteristics, and the types of outcomes emphasized. Context was therefore used primarily to qualify cross-study comparisons and the interpretation of outcome patterns, rather than to treat different settings as directly comparable intervention conditions (see Table 3).

**Table 3:** Implementation context and professional practice background of the included studies.

Implementation context/professional practice background	Practitioner professional background	Implementation intensity	Learner characteristics	Outcomes primarily emphasized	Corresponding studies
Mainstream classroom contexts	Practitioners typically had backgrounds in classroom teaching, language and literacy education, music or arts education, or multimodal pedagogy; some programmes were implemented collaboratively by teachers and researchers.	Ranged from single-session or short-term classroom activities to programmes extending across several weeks or semesters, typically embedded within regular instruction or school-based learning activities.	School-aged children in mainstream education, including language learners, immigrant children, and students from diverse sociocultural backgrounds.	Vocabulary, reading, comprehension, narrative expression, learning participation, creative expression, and identity-related meaning-making.	S3, S7, S13, S15, S17, S18
Early-childhood education contexts	Practitioners mainly included early-childhood educators, educational researchers, school-based professionals, and practitioners with experience in child development; caregivers were involved in some studies.	Ranged from brief experimental book-sharing activities to structured programmes delivered repeatedly over several weeks, including repeated reading, singing, and embodied activities.	Primarily preschool and younger children, including typically developing children and children with different communicative or developmental profiles.	Vocabulary, story comprehension, attention, emergent literacy, socioemotional development, embodied participation, and interaction.	S1, S4, S6, S11, S12, S14, S17
Family and community contexts	Practitioners included parents, family members, community participants, Indigenous educators or Elders, and researchers, with greater emphasis on family knowledge and culturally situated community practices.	Typically involved repeated participation over several weeks or longer periods through family-based activities, shared practices, or sustained community collaboration.	Preschool or school-aged children participating with their families, including children from specific linguistic, cultural, or Indigenous communities.	Early language and literacy, shared participation, family interaction, cultural identity, cultural continuity, and relational meaning-making.	S8, S19
Inclusive education contexts	Practitioners mainly included educational researchers, teachers or facilitators, and caregivers, with practices adapted to children’s nonverbal communication, embodied expression, and developmental differences.	Included both repeated activities and longitudinal observation in naturalistic educational settings, as well as structured parent–child spoken or sung book-sharing tasks.	Primarily autistic children and children with differing communicative, linguistic, or developmental characteristics.	Communication and participation, shared attention, emotional regulation, embodied expression, social interaction, and social synchrony.	S6, S14
Therapeutic and supportive programmes	Practitioners mainly included music therapists, arts therapists, trained facilitators, educators, and other professionals working in psychosocial, trauma-informed, bereavement, or relational-support contexts.	Typically involved repeated and structured sessions or ongoing therapeutic/supportive processes, ranging from a series of workshops to longer-term case-based or programme-based work.	Children experiencing bereavement, trauma, migration or refugee experiences, emotional difficulties, or relational stress.	Emotional expression and regulation, safety, self-soothing, agency, belonging, social connection, and relational repair.	S2, S5, S9, S10, S16

### 4.2 Outcome evidence and process-related affordances

For RQ2, evidence was distinguished between developmental outcomes and process-, participation-, or expression-related affordances. Developmental outcomes referred to measured or clearly documented changes in children’s language–narrative or socioemotional functioning, whereas process-, participation-, and expression-related affordances referred to observed or reported opportunities for participation, expression, agency, interaction, or meaning-making that did not themselves constitute evidence of developmental change.

#### 4.2.1 Language–narrative outcomes and expressive affordances

The available evidence indicates heterogeneous findings regarding language–narrative development in music-supported narrative learning. Positive changes in vocabulary, reading, and narrative performance co-occurred across studies with small, uncertain, or occasionally negative effects, reflecting diverse outcome patterns rather than a consistent overall effect.

Several quantitative studies reported changes in vocabulary and reading performance, although their magnitude varied. In S17, children in the shared-singing group showed higher immediate recall of target vocabulary than those in the shared-reading group (*M* = 6.11 vs. 4.87, *d* = 0.41), with a similar pattern observed for delayed retention 16 days later (*M* = 6.04 vs. 4.89, *d* = 0.39). In S7, word-reading fluency changed from 79.3 to 91.8%, while sentence-reading automaticity increased by an average of 30.6 and 35.2 words per minute for narrative and expository texts, respectively. Pre–post changes in reading prosody were large (narrative text: *d* = 2.30, 95% CI [1.58, 4.65]; expository text: *d* = 2.02, 95% CI [1.51, 3.68]), whereas narrative reading accuracy changed only from 96.6 to 97.0%. In S8, children were unable to correctly name any of the eight target words at baseline but named an average of four after completing the relevant programme component, and this vocabulary change was maintained at the six-month follow-up.

In other multimodal practices, measurable changes co-occurred with broader instructional designs. In S15, the mean English test score changed from 0.26 to 0.51 (an increase of 0.24), but the programme simultaneously integrated narrative, programming, images, animation, English reading aloud, recording, and sound editing. In S18, the authors reported improvements in performance literacy and digital storytelling/filmmaking, with the quality of digital storytelling/filmmaking increasing by an average of 2.00 points on a 10-point scale. Improvements were also reported in story elements, science vocabulary, writing, oral fluency, and narration. Standardized effect sizes or precision estimates were not reported. These data therefore reflect changes within integrated multimodal learning environments, and the available evidence is insufficient to isolate the independent contribution of music-related activities.

Qualitative and participatory studies provided a different type of evidence. Children expressed personal experiences, identities, cultural knowledge, and emotions through lyrics, singing, movement, drawing, sound, digital media, and recording. Songwriting in S2 revealed distinct lyrical themes related to bereavement experiences, memories, and understandings of death, with older participants producing more diverse lyrical content; S6 showed that movement, spontaneous vocalization, and rhythm could provide ways for children with limited spoken language to participate in narrative activities; and S13 and S19 further showed that music and other multimodal resources could be embedded within narrative expression related to names, culture, and identity. However, these findings primarily reflect process- and expression-related affordances.

At the same time, some studies did not show clear learning advantages. In S1, story-comprehension scores were similar between conditions with narratively congruent music and environmental sounds and those without music/sounds (adjusted *M* = 17.36 vs. 17.53). Receptive-vocabulary scores were lower when music/sounds were present (adjusted *M* = 16.52 vs. 18.86, *d* = −0.56), while expressive vocabulary showed no clear difference. In S4, a single auditory or visual enhancement was associated with slightly higher story-comprehension scores than combined enhancements (*M* = 9.45 vs. 8.72; effect size = 0.16), whereas no corresponding difference was observed for vocabulary. Overall, the available evidence suggests that the functional integration of music or sound with narrative content was not consistently associated with stronger language–narrative outcomes.

#### 4.2.2 Socioemotional outcomes and relational affordances

Socioemotional evidence primarily concerned emotional expression and regulation, self-understanding and agency, and social connection and belonging.

Regarding emotional expression and regulation, several qualitative studies reported similar process patterns: children expressed experiences and emotions that were difficult to communicate directly through lyrics, sounds, movement, role-play, drawing, and recording, while singing, rhythm, repetition, breathing, and bodily activities frequently co-occurred with safety, relaxation, self-soothing, emotional openness, and sustained participation (S5, S6, S9, S10, S16).

Quantitative evidence was reported in S11. Following a programme integrating music, stories, yoga, breathing, and massage, the programme group showed higher social-competence scores than the comparison group (*M* = 43.35 vs. 40.94, *d* = 0.52), lower anger–aggression scores (*M* = 15.67 vs. 17.16, *d* = 0.78), and higher psychosocial-health scores (*M* = 83.87 vs. 78.65, *d* = 0.50). By contrast, estimated between-group effects were close to zero for emotional stability/stress coping (*η*^2^ = 0.000) and anxiety–introversion (*η*^2^ = 0.003).

Regarding self-understanding and agency, personalized songs, name-related narratives, digital products, and recording activities were more frequently reported in contexts involving identity, cultural belonging, ownership, perceived control, and self-expression (S3, S9, S13, S15, S16, S19). For example, in S13, children used percussion and generative songs to express their names and associated cultural meanings, while also showing interest in, pride toward, and reflection on their name stories and creative products. In the single-case study S16, the recording process co-occurred with agency, memory integration, and reconstruction of the parent–child relationship, although these findings were clearly case- and context-dependent.

Regarding social connection, music-supported narrative learning practices involving shared singing, songwriting, drawing, story sharing, and collaborative recording frequently co-occurred with peer relationships, group belonging, shared attention, and relational participation (S2, S3, S5, S11, S12, S13, S14, S16). Qualitative evidence from S12 showed that, during collaborative creation, drawing, and narrative activities, children negotiated group roles, expressed peer acceptance, and constructed shared experiences, although these interactions could also make pre-existing peer tensions more visible. Quantitative evidence from the parent–child context in S14 showed a more limited pattern: visual attention was higher in the sung book-sharing condition than in the spoken condition for both children (*M* = 2.19 vs. 2.07, generalized *η*^2^ = 0.05) and caregivers (*M* = 3.73 vs. 3.33, generalized *η*^2^ = 0.16), whereas the effect of book type on parent–child movement coordination was small (generalized *η*^2^ = 0.01). Overall, the available evidence suggests that process-level manifestations of social connection and relational participation were relatively consistently observed in music-supported narrative learning practices, but these manifestations did not always correspond to measurable changes in social interaction.

### 4.3 Cross-study patterns related to differences in reported outcomes

Cross-study analyses revealed several recurring patterns involving instructional design, participation structure, learner characteristics, and implementation context. On the basis of these cross-study comparisons, two broader interpretive patterns were further identified. The information-organization pattern refers to recurring configurations in which rhythm, melody, singing, or sound was functionally aligned with target words, plot events, narrative sequences, or other task-relevant information and was primarily associated with language–narrative outcomes. The expression–relationship pattern refers to recurring configurations in which generative, collaborative, or child-led participation co-occurred with personal expression, identity, agency, belonging, or relational participation. These two patterns were derived through cross-study comparison of the practice-feature–outcome matrix developed from the RQ1 and RQ2 syntheses and therefore summarize recurring configurations in the evidence. They should therefore be interpreted as patterns of co-occurrence across heterogeneous studies rather than as causal effects of individual practice characteristics. Figure 3 visually summarizes the practice features, learner characteristics, and implementation conditions discussed in Section 4.3 in relation to the outcome patterns with which they co-occurred. The connections shown in the figure represent cross-study relationships used for explanatory synthesis, not tested causal pathways or mediation mechanisms (see Figure 3).

**Figure 3:**
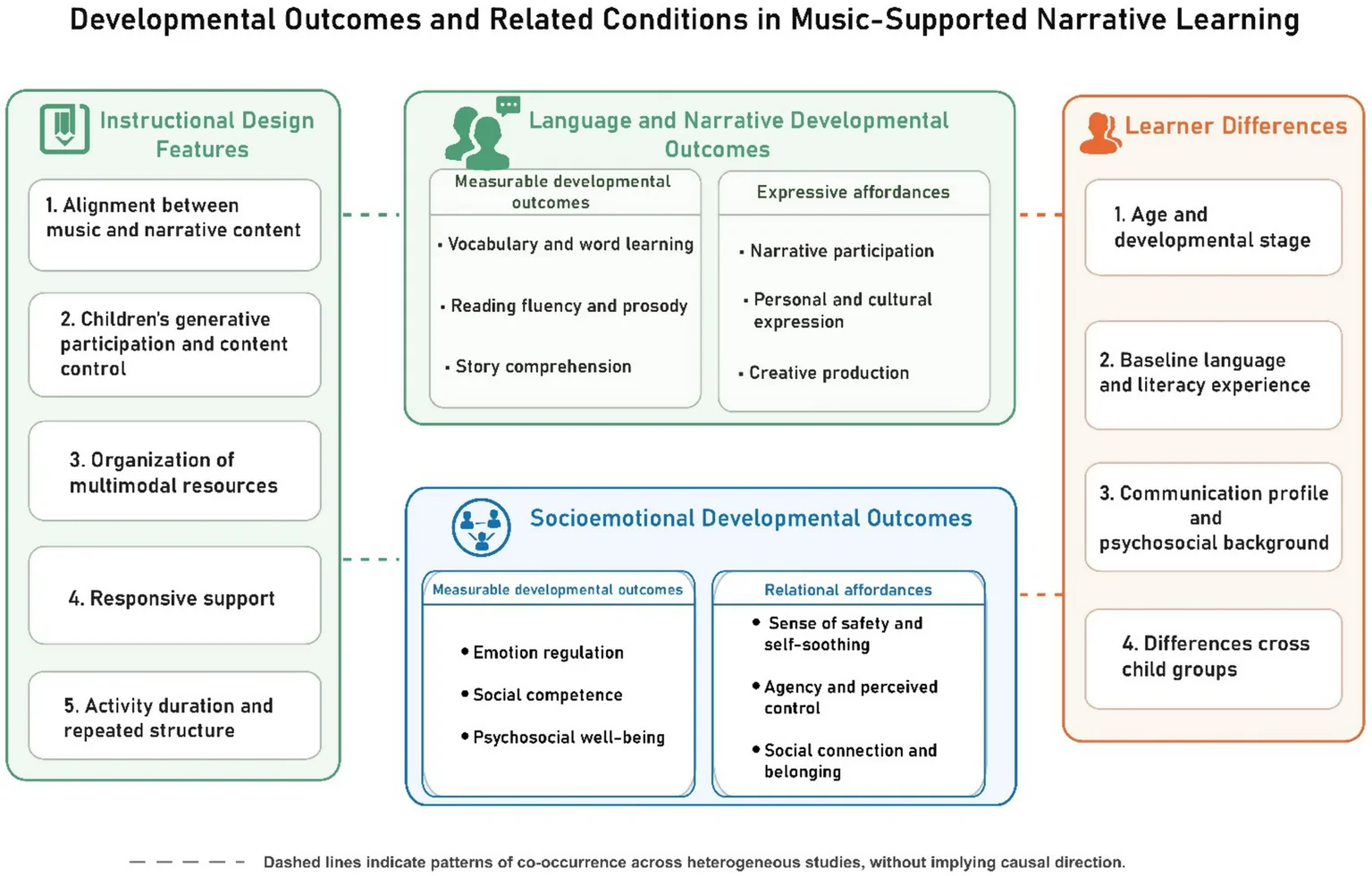
Developmental outcomes and related conditions in music-supported narrative learning.

#### 4.3.1 Instructional design features

First, one recurring pattern concerned the functional relationship between musical materials and narrative tasks. When rhythm, melody, singing, or sound was organized around narrative content or target language material, the corresponding studies more frequently reported positive changes in language learning. For example, in S7, sentence-reading automaticity increased by 30.6–35.2 words per minute, with pre–post changes in reading prosody of *d* = 2.02–2.30. In S17, shared singing showed small-to-moderate advantages for immediate and delayed vocabulary performance (*d* = 0.41 and 0.39, respectively). However, functional alignment did not consistently correspond to positive outcomes. In S1, narratively congruent music and environmental sounds were associated with virtually no difference in story comprehension, while receptive-vocabulary performance was lower (*d* = −0.56).

Second, different participation structures co-occurred with different types of evidence. Receptive practices based primarily on listening and viewing more frequently examined immediate comprehension, vocabulary, and attention (S1, S4, S14), whereas collaborative and child-led practices more commonly reported personal narrative, emotional expression, identity, agency, ownership, and belonging (S2, S3, S5, S6, S9, S10, S13, S15, S16, S18, S19). Evidence related to agency and identity was also more frequently reported in studies in which children made decisions about lyrics, narrative themes, emotional content, or the direction of product revision.

Third, different patterns were observed in the organization of multimodal resources. The simultaneous use of multiple modalities was not consistently associated with stronger learning outcomes. In S4, story-comprehension scores were slightly higher with a single auditory or visual enhancement than with combined enhancements (*M* = 9.45 vs. 8.72; effect size = 0.16), and this pattern was particularly evident among younger children. By contrast, practices that organized listening, responding, creating, and sharing into sequential phases more frequently co-occurred with narrative expression, identity exploration, emotional expression, and collaborative meaning-making (S5, S10, S12, S15, S18, S19).

Finally, responsive support and sustained participation were also recurring practice characteristics. In studies in which adults adapted activities in response to children’s movements, vocalizations, emotions, or creative choices, sustained attention, emotional openness, and a sense of control were more frequently reported (S6, S10, S16). Repeated and sustained programmes also more commonly reported reading development, personal narrative, emotional participation, and relational outcomes (S2, S3, S5, S7, S8, S17). Programme duration and repetition, however, varied together with programme purpose, professional practice background, and implementation format.

#### 4.3.2 Learner differences

Age was one of the few learner characteristics examined directly. In S4, outcome patterns associated with single versus combined multimedia enhancements varied by age, with the clearest advantage for a single enhancement observed among three-year-old children. Songwriting research also showed that older children and adolescents produced more diverse lyrical themes and expressed more complex personal experiences (S2).

Patterns related to baseline language and literacy experience were less consistent. In S7, children with different levels of Korean-language exposure and different starting levels showed substantial increases in reading automaticity, but the magnitude of improvement did not vary consistently with language background. For children with more limited spoken language, movement, sound, and other nonverbal modes provided additional forms of narrative participation (S6, S12). In S14, language level was strongly positively associated with visual attention among autistic children (*r*_s_ = 0.67), whereas the same pattern was not observed among typically developing children.

Children’s developmental, psychosocial, and sociocultural backgrounds also varied with the types of evidence emphasized across studies. Studies involving children experiencing trauma, bereavement, or emotional difficulties more frequently reported the expression of difficult emotions, safety, perceived control, shared remembering, and relational repair (S2, S9, S10, S16). Studies involving autistic children more commonly focused on multimodal communication, embodied participation, attention, and interaction (S6, S14). Studies involving immigrant or refugee children more frequently emphasized second-language learning, cultural connection, belonging, and emotional expression (S5, S7). Studies involving Indigenous or culturally situated learning more commonly emphasized language and literacy, cultural identity, cultural continuity, and family or community participation (S8, S19).

## 5 Discussion and conclusion

### 5.1 Key findings and explanatory patterns

This review indicates that the outcomes and affordances reported in music-supported narrative learning cannot be explained simply by whether music was included. Rather, the reported patterns varied with the function of music within the task, the ways in which children participated, and their position in the process of meaning-making. When musical materials were functionally organized around target language or narrative content, some studies reported more favorable language–narrative outcomes, although this pattern was not consistent. Supplementary or background music, by contrast, was associated with limited, mixed, and occasionally negative findings. More generative music-supported narrative learning practices involving songwriting, movement, drawing, and recording more frequently co-occurred with process-, expressive-, and relational affordances involving emotional expression, identity, agency, and social connection.

These patterns are broadly consistent with the central propositions of narrative development theory and sociocultural learning theory, according to which the educational significance of music is related to how it participates in narrative organization, linguistic expression, interactional support, and shared meaning-making rather than being inferred from the presence of music alone (Pico et al., 2021; Blasco-Magraner et al., 2021). Accordingly, the functional relationship between music and the narrative task and children’s participatory position within the activity provide two useful dimensions for understanding cross-study differences.

The heterogeneity of language–narrative outcomes is also consistent with the boundary explanations provided by cognitive load and task alignment. Alignment between music and content did not itself guarantee a learning advantage, while background auditory information could interfere with comprehension under some conditions, particularly when it contained distracting lyrical or acoustically demanding features (Ba and Hu, 2025; Que and Hu, 2025). By comparison, the reading and language changes reported following structured singing occurred directly in relation to target-language material and may therefore, to some extent, be understood as within-task scaffolding or near transfer to the target material rather than broad cross-domain cognitive transfer.

In the socioemotional domain, measured developmental outcomes were not distributed in the same way as process-level affordances across practices. Activities that allowed children to incorporate personal experiences, choose lyrics or topics, and participate in revising products more frequently reported evidence related to agency, identity, emotional expression, and belonging. This pattern is consistent with self-determination theory’s emphasis on autonomy, competence, and relatedness (Ryan and Deci, 2000). However, the available evidence primarily indicates co-occurrence between children’s control over content and these process-level manifestations, describing associations rather than establishing autonomous control as an independent causal mechanism. Similarly, social connection and relational participation observed in shared musical activities are broadly consistent with previous research emphasizing social interaction and group affiliation in joint music-making (Papageorgi et al., 2022). Within the present review, these manifestations are more appropriately characterized as relational affordances arising within particular interaction structures.

From the perspective of psychological regulation, several included studies repeatedly reported process-level manifestations involving safety, relaxation, self-regulation, sustained attention, and emotional openness (Mohammadhosseini and Schmid, 2025; Zacharová and Liliková, 2026; Hadar, 2025). These observations are consistent with the temporal organization, affective processing, embodied participation, and shared interaction processes outlined in the theoretical framework. Some music-supported narrative learning practices also explicitly incorporated breathing, movement, rhythmic coordination, or synchronized musical activity (Mohammadhosseini and Schmid, 2025; Zacharová and Liliková, 2026; Algül and Kılıçarslan, 2026). Bodily arousal and interpersonal coordination may represent candidate processes for further investigation. However, few included studies directly measured heart rate, electrodermal activity, or respiratory change, and the available evidence therefore primarily supports psychological and behavioral interpretations. Future research could combine self-report, behavioral observation, and low-burden physiological measures to examine whether immediate regulation during activities is associated with more stable socioemotional change. The transferability of these processes across settings remains uncertain. Therapeutic and supportive programmes were often delivered by trained professionals, involved children with specific psychosocial needs, and provided more intensive or individualized support than is typical in mainstream classrooms. Processes such as psychological safety, self-soothing, relational repair, or emotional disclosure may therefore depend partly on therapeutic relationships and implementation conditions rather than on the musical activity itself. Accordingly, similar practices should be examined directly in mainstream educational settings before assuming that the same processes or outcomes will occur.

Cross-study coding also revealed substantial structural clustering. Learner characteristics, practice structures, programme purposes, and outcome measures frequently occurred in particular combinations rather than being examined independently. Consequently, although the literature shows several recurring patterns of co-occurrence, it remains difficult to distinguish whether these differences are associated with learner characteristics, practice design, or alignment between the two. Few studies directly compared children differing in age, linguistic background, neurodevelopmental characteristics, or psychosocial experience under the same music-supported narrative learning practice, implementation intensity, and measurement conditions. Studies also rarely examined language–narrative outcomes, socioemotional outcomes, and process-level participation within the same design.

Such structural clustering may reflect a combination of learner needs, practice design, research traditions, and measurement choices. Because children’s narrative performance may itself be jointly associated with age, language experience, and task complexity (Lipner et al., 2024), cross-study differences cannot currently be attributed to any single characteristic. Future research could employ a common set of core outcomes under comparable conditions and directly examine interactions between practice characteristics and learner characteristics, helping to distinguish design principles with broader applicability from implementation conditions requiring adaptation for particular learners. More importantly, these recurring cross-study patterns should be treated as emerging patterns requiring direct future testing, rather than as established mechanistic relationships.

Finally, the socioemotional evidence showed some outcome specificity. Some programmes reported changes in participation, relational interaction, psychological resilience, or social competence but did not show consistent changes in broader measures such as anxiety or stress coping. This pattern bears some similarity to the limited and uneven findings reported in parts of the music therapy literature by Ke et al. (2022). The available evidence therefore supports distinguishing proximal changes in participation, expression, and interaction within the activity context from broader and more stable developmental outcomes; the former should not automatically be treated as evidence of the latter.

### 5.2 Developmental stages and outcome patterns

The included studies showed some variation in the forms and reported outcomes of music-supported narrative learning across developmental stages. In preschool research, music was more often embedded as an external scaffold within picture-book, story-comprehension, and early-language activities, with greater emphasis on attention, vocabulary retention, story comprehension, and emergent literacy. When multiple visual and auditory enhancements were presented simultaneously, younger children showed less favorable learning outcomes under some conditions, a pattern consistent with greater sensitivity to additional processing demands (Li and Bus, 2023). Continuous background music without a direct relationship to the narrative likewise showed no clear learning advantage in related studies and was sometimes associated with weaker vocabulary performance (Sarı et al., 2019).

During the primary-school years, music-supported narrative learning practices more frequently involved songwriting, personal narrative, movement, drawing, digital creation, and collaborative expression. The corresponding evidence concerned not only linguistic expression but also expressive or relational affordances involving personal experience, identity, agency, and group belonging (Dweck, 2024). Studies at this stage more frequently reported processes in which children generated content, negotiated meaning, and expressed personal or social experiences through music and other modalities.

Age, however, may not be the only learner characteristic associated with these patterns. Language experience, communicative ability, prior literacy experience, initial interest, and socioemotional needs may also be related to the forms of participation available to children. For example, language-learning changes among immigrant children may be associated with length of residence, home language, and exposure to the target language (Choe et al., 2025). The distinction between preschool and primary-school research should therefore not be treated as a fixed developmental boundary. Rather, the current literature suggests a possible shift from greater use of music to support story comprehension and language processing toward broader use of music for narrative generation, personal expression, and the construction of identity- and relationship-related meaning.

Cultural context represents another source of variation that cannot be clearly separated in the current evidence. The included studies were conducted across different national, linguistic, and cultural settings, and several practices explicitly incorporated names, migration experiences, local traditions, family narratives, or Indigenous cultural knowledge (Derby, 2023; Lin et al., 2022; Mills et al., 2025; Mohammadhosseini and Schmid, 2025; Yang and Harper, 2026). Such content is not culturally interchangeable. The meanings attached to musical materials, acceptable forms of emotional expression, patterns of adult–child interaction, and opportunities for child agency may vary across cultural settings. Accordingly, the two interpretive patterns identified in this review should not be assumed to generalize uniformly across cultures. Future research should examine whether and how music-supported narrative practices need to be adapted to local languages, musical traditions, family and community expectations, and culturally specific forms of narrative meaning-making.

This developmental pattern nevertheless requires cautious interpretation. Preschool studies more often employed experimental digital picture-book or structured language tasks, whereas studies with primary-school children more frequently used qualitative, creative, collaborative, or supportive practices. Age, activity type, study design, learner background, and outcome measurement were therefore substantially structurally clustered, and current cross-study differences cannot be attributed to age itself. Future research should directly compare children of different ages under similar music-supported narrative learning practices, implementation intensities, and measurement conditions to examine the relationship between developmental stage and practice characteristics more directly.

### 5.3 Limitations

This review has several limitations. First, the review protocol was not prospectively registered or publicly archived. The review was initially conducted as an investigator-led, unfunded project, and prospective registration was not completed before the screening process began. We acknowledge that the absence of prospective registration limits independent verification of whether methodological decisions changed during the review process and may increase the possibility of unrecognized post-hoc methodological decisions. To improve transparency, the final database search strategies, eligibility criteria, reviewer roles, criterion-level MMAT appraisal, and completed PRISMA 2020 checklist are provided in the Supplementary material.

Second, the search was restricted to English-language publications, and the number of eligible studies was relatively limited, which may restrict the coverage and comparability of the evidence. The included evidence did not show a uniformly positive pattern: favorable findings co-occurred with null, small, uncertain, and occasionally negative findings, sometimes within the same study. Nevertheless, publication bias remains possible because studies reporting weak or null effects may be less likely to be published or easily identified. The recurring patterns identified in this review may therefore overrepresent practices associated with favorable outcomes and should not be interpreted as evidence of consistently beneficial effects.

Third, the included evidence comprised randomized trials, non-randomized studies, mixed-methods studies, and qualitative and case-based research. Although methodological quality was assessed using the MMAT, substantial variation remained in study design, sample characteristics, practice format, implementation intensity, and outcome measurement. These differences limited direct comparison across studies, and the relationships identified in this review should therefore primarily be interpreted as patterns of co-occurrence across heterogeneous studies.

Fourth, some studies used multicomponent designs combining music with visual arts, movement, yoga, digital technologies, or other activities. Although music had a substantive functional relationship with an identifiable narrative process, the independent contribution of music-related activity could not always be separated from the broader instructional or supportive design.

Finally, part of the evidence was derived from inclusive education, family/community, and therapeutic or supportive contexts that differed from mainstream classrooms in professional practice background, programme purpose, implementation intensity, and learner needs. At present, there is insufficient direct evidence that practices identified in these contexts produce comparable outcomes when transferred to mainstream classrooms. They should therefore be treated as hypotheses for future educational testing rather than as established design recommendations. Findings concerning safety, self-soothing, agency, or relational repair may inform hypotheses involving responsive support, multimodal expression, and learner agency, but should not be used to infer equivalent effects or developmental outcomes in mainstream educational settings without direct examination.

### 5.4 Conclusions and contribution

Overall, the main contribution of this review is not to demonstrate that music broadly promotes child development, but to bring language–narrative and socioemotional evidence into a common framework and clarify how practice characteristics are related to different types of outcomes within music-supported narrative learning. The synthesis revealed two interrelated emerging interpretive patterns, an information-organization pattern and an expression–relationship pattern. The former concerns the functional relationship between music and vocabulary, plot events, and narrative sequences, whereas the latter reflects the co-occurrence of generative and shared practices with personal expression, identity, agency, and relational participation. Given the limited evidence base and substantial structural clustering, these patterns should be regarded as interpretive propositions requiring further testing. Future studies could examine, under more comparable learner populations, practice conditions, and outcome measures, the relationship between music–narrative information organization and language–narrative outcomes, as well as the relationship between children’s participation and control over content and expressive, agentic, and relational affordances, to determine whether these emerging patterns can be replicated across contexts.

The contribution of this framework lies in moving the field beyond the general question of whether music “works” toward examining how musical function, participation structure, and learner conditions combine within different practice configurations. Music-supported narrative learning is therefore better understood not as a uniform pedagogical format, but as a set of practices whose interpretation depends on specific task goals, participation structures, and learner needs. This perspective provides a more structured basis for understanding inconsistencies across the existing literature and offers directions for developing more comparable and testable research designs.

## 6 Implications for curriculum and practice

Given the limited evidence base, methodological heterogeneity, and structural clustering among practice characteristics, learner populations, and implementation contexts, the following implications are offered as exploratory considerations for curriculum design and practice hypotheses requiring further testing, particularly from the perspectives of relational affordances and contextual alignment.

First, future curriculum research could examine whether music-supported narrative learning practices are more effective when organized around clearly specified learning or developmental goals, with participation structures and levels of support adapted to children’s language experience, communicative characteristics, and socioemotional needs, rather than by simply adding music to existing narrative activities or organizing activities solely by age.

Second, when the goals concern vocabulary, story comprehension, or reading, future practice and research may further examine the functional alignment of musical materials with target words, key plot events, and other task-relevant information, while using continuous background music without a clear relationship to the learning goal with caution (Zhou and Li, 2017). Future research should examine whether the relative value of multimodal alignment and generative tasks varies across developmental stages under comparable implementation and measurement conditions.

When the focus shifts toward emotional expression, identity, belonging, or social relationships, future studies could examine whether practices that provide greater opportunities for children’s content choice, collaborative creation, and multimodal expression are associated with stronger expressive or relational affordances (Fiore, 2016). Movement, drawing, improvised sound, and recording may offer additional routes for participation for children with differing verbal or communicative profiles. Practices derived from therapeutic or specialist support settings, however, should not be treated as evidence of equivalent effects in mainstream classrooms; their applicability needs to be considered in relation to practitioner expertise, implementation conditions, and learner needs.

At the curriculum-implementation level, music-supported narrative learning practices could be further explored as combinable design modules, including language-information organization, personal-experience expression, peer collaboration, and multimodal support, with adaptation to target learners and instructional conditions. Sustained and sequenced formats also warrant further comparison, although the present evidence is insufficient to prescribe a uniform duration or fixed model. These design directions are therefore more appropriately treated as objects for direct testing in future curriculum research. Their practical feasibility should also be considered, as collaborative creation, digital production, recording, movement, or individualized support may require additional teacher preparation, instructional time, equipment, space, and financial resources (Stanley, 2021). Future implementation research should therefore examine not only developmental outcomes, but also teacher workload, resource requirements, feasibility, and sustainability in everyday educational settings.

## Data Availability

The original contributions presented in the study are included in the article/Supplementary material, further inquiries can be directed to the corresponding author.
