# Supplementary material for: When music tells a story: children’s language and socioemotional development through music-supported narrative learning
Source: Front Psychol. 2026 Sep 17;17:1956337. doi: 10.3389/fpsyg.2026.1956337 (PMC13627311; doi:10.3389/fpsyg.2026.1956337)
Supplement: Supplementary file 1 [file Supplementary_file_1.docx]

**Table 1. Database-Specific Search Strategies**

| Database | Search field | Complete search strategy | Limits | Final search date |
| --- | --- | --- | --- | --- |
| Scopus | TITLE-ABS-KEY | TITLE-ABS-KEY((music OR sing OR song OR rhythm* OR melody OR melodic) AND (educat* OR teach* OR learn* OR instruct* OR pedagog* OR curriculum OR classroom* OR school* OR lesson* OR training OR program* OR intervention*) AND (narrativ* OR storytell* OR storybook* OR "picture book*" OR "story-based learning" OR "story-based teaching" OR songwriting OR "lyric writing" OR "music drama" OR "musical drama" OR "role play*" OR "story creation" OR "story composition" OR "story retelling") AND ((wellbeing OR "well-being" OR socioemotional OR "social-emotional" OR "social emotional" OR "emotional development" OR "emotion regulation" OR "emotional competence" OR "emotional expression" OR resilience OR "self-esteem" OR confidence OR belonging OR prosocial* OR "social competence" OR "psychological wellbeing" OR "psychological well-being") OR ("language development" OR linguistic* OR vocabulary OR literacy OR reading OR writing OR "oral language" OR "spoken language" OR "communication skill*" OR "narrative skill*" OR "narrative competence" OR "narrative ability" OR "storytelling skill*" OR "expressive language" OR "receptive language" OR comprehension OR "verbal ability"))) | English; article; publication period 2016–2026 | August 22, 2026 |
| Web of Science Core Collection | Topic | TS=((music OR sing OR song OR rhythm* OR melody OR melodic) AND (educat* OR teach* OR learn* OR instruct* OR pedagog* OR curriculum OR classroom* OR school* OR lesson* OR training OR program* OR intervention*) AND (narrativ* OR storytell* OR storybook* OR "picture book*" OR "story-based learning" OR "story-based teaching" OR songwriting OR "lyric writing" OR "music drama" OR "musical drama" OR "role play*" OR "story creation" OR "story composition" OR "story retelling") AND ((wellbeing OR "well-being" OR socioemotional OR "social-emotional" OR "social emotional" OR "emotional development" OR "emotion regulation" OR "emotional competence" OR "emotional expression" OR resilience OR "self-esteem" OR confidence OR belonging OR prosocial* OR "social competence" OR "psychological wellbeing" OR "psychological well-being") OR ("language development" OR linguistic* OR vocabulary OR literacy OR reading OR writing OR "oral language" OR "spoken language" OR "communication skill*" OR "narrative skill*" OR "narrative competence" OR "narrative ability" OR "storytelling skill*" OR "expressive language" OR "receptive language" OR comprehension OR "verbal ability"))) | English; article; publication period 2016–2026 | August 22, 2026 |
| ERIC | / | (music OR sing OR song OR rhythm* OR melody OR melodic) AND (educat* OR teach* OR learn* OR instruct* OR pedagog* OR curriculum OR classroom* OR school* OR lesson* OR training OR program* OR intervention*) AND (narrativ* OR storytell* OR storybook* OR "picture book*" OR "story-based learning" OR "story-based teaching" OR songwriting OR "lyric writing" OR "music drama" OR "musical drama" OR "role play*" OR "story creation" OR "story composition" OR "story retelling") AND ((wellbeing OR "well-being" OR socioemotional OR "social-emotional" OR "social emotional" OR "emotional development" OR "emotion regulation" OR "emotional competence" OR "emotional expression" OR resilience OR "self-esteem" OR confidence OR belonging OR prosocial* OR "social competence" OR "psychological wellbeing" OR "psychological well-being") OR ("language development" OR linguistic* OR vocabulary OR literacy OR reading OR writing OR "oral language" OR "spoken language" OR "communication skill*" OR "narrative skill*" OR "narrative competence" OR "narrative ability" OR "storytelling skill*" OR "expressive language" OR "receptive language" OR comprehension OR "verbal ability")) | English; peer-reviewed; publication period 2016–2026 | August 22, 2026 |

**Table 2. Reviewer Roles and Reliability Procedures Across Review Stages**

| Stage | Reviewer 1 | Reviewer 2 | Reliability and disagreement procedure |
| --- | --- | --- | --- |
| Eligibility criteria and pilot screening | Jointly developed and applied the criteria | Jointly developed and applied the criteria | Screening rules and operational boundaries were clarified through discussion |
| Title and abstract screening | Screened all records | Independently screened a randomly selected 20% subset | Cohen’s *κ* = .87; disagreements were resolved through record re-examination and discussion |
| Remaining 80% of titles and abstracts | Completed screening | Reviewed uncertain cases | Uncertain records were retained for joint discussion rather than excluded by one reviewer alone |
| Full-text screening (26 reports) | Independently assessed all full texts | Independently assessed all full texts | Pre-consensus agreement = 92.3%; inclusion/exclusion decisions and reasons were compared, and disagreements were resolved through full-text re-examination and consensus discussion |
| Descriptive data extraction | Extracted all study information | Checked all extracted information | Discrepancies were corrected through direct verification against the full text |
| Interpretive coding of practice characteristics | Independently coded all included studies | Independently coded all included studies | Pre-consensus agreement = 90%; disagreements concerning category assignment or coding boundaries were resolved through re-examination of the original studies and discussion |
| Outcome-category coding | Independently coded all included studies | Independently coded all included studies | Pre-consensus agreement = 88%; disagreements concerning developmental versus process-, participation-, or expression-related evidence were jointly reviewed and resolved |
| Thematic synthesis and cross-study interpretive matrix | Developed the initial thematic structure and matrix | Independently reviewed themes, category boundaries, and study assignments | Discrepant theme or study assignments were re-examined against the consensus-coded data and jointly refined |
| MMAT quality appraisal | Independently appraised all included studies | Independently appraised all included studies | Pre-consensus item-level agreement = 91%; disagreements were resolved by re-examining the methodological information and reaching consensus |

**Table 3. Methodological Quality Appraisal of the Included Studies Using the MMAT**

| No. | Study | MMAT category | Q1 | Q2 | Q3 | Q4 | Q5 |
| --- | --- | --- | --- | --- | --- | --- | --- |
| 1 | A randomized controlled trial to test efficacy of digital enhancements of storybooks in support of narrative comprehension and word learning | Randomized controlled trial | Y | Y | Y | CT | Y |
| 2 | Analysis of Lyrics from Group Songwriting with Bereaved Children and Adolescents | Mixed methods | Y | CT | CT | CT | CT |
| 3 | Children as songwriters: The social-emotional benefits of songwriting in the elementary grades | Qualitative | Y | Y | Y | Y | Y |
| 4 | Efficacy of digital picture book enhancements grounded in multimedia learning principles: Dependent on age? | Randomized controlled trial | Y | Y | Y | CT | Y |
| 5 | Embodied engagement: Fostering caring thinking in a multimodal singing-based learning environment for refugee children | Qualitative | Y | Y | Y | Y | Y |
| 6 | Embodied narrative learning as an inclusive practice in early childhood settings: Rhythm, movement, and emotional engagement in children with autism spectrum disorder | Qualitative | Y | Y | Y | Y | Y |
| 7 | Enhancing text-level reading fluency and engagement in immigrant children through a structured singing-based intervention | Mixed methods | Y | Y | Y | CT | CT |
| 8 | Talking Together: The Effects of Traditional Māori Pedagogy on Children’s Early Literacy Development | Non-randomized study | CT | Y | Y | CT | Y |
| 9 | I Will Follow You: The Combined Use of Songwriting and Art to Promote Healing in a Child Who Has Been Traumatized | Qualitative | Y | CT | Y | Y | Y |
| 10 | Integration of music, yoga and massage: Investigation of their effects on preschool children’s social-emotional development and quality of life | Non-randomized study | CT | Y | Y | CT | Y |
| 11 | ‘It Is You in My Picture!’ A Holistic Approach to Six-Year-Old Children’s Art Experiences | Qualitative | Y | Y | Y | Y | Y |
| 12 | “I didn’t know I have the capacity to be creative”: Children’s experiences of how creativity promoted their sense of well-being. A pilot randomised controlled study in school arts therapies | Mixed methods | Y | CT | Y | CT | CT |
| 13 | Names Matter: Fostering Cultural Awareness Through Multimodal Literacy in a Predominantly White Classroom | Qualitative | CT | N | N | CT | CT |
| 14 | Parent-child nonverbal engagement during spoken versus sung book-sharing in preschoolers with and without autism | Non-randomized study | CT | Y | Y | Y | Y |
| 15 | Programming E-Books: Culture, English, and Scratch for Schoolchildren of Rural Taiwan | Non-randomized study | CT | CT | Y | N | Y |
| 16 | Relational repair through recording: A music therapy case study with a child and parents following the October 7 attack | Qualitative | Y | Y | Y | Y | Y |
| 17 | The Effects of Shared Singing Picture Book Instruction on Chinese Immersion Kindergarteners’ Spoken Vocabulary Recall and Retention | Randomized controlled trial | Y | CT | Y | CT | Y |
| 18 | Poetry and digital media for improving upper elementary African American science learning | Qualitative | Y | Y | CT | CT | Y |
| 19 | Multimodal Language Rights and Indigenous Totemic Identity: Cross-Cultural Participatory Research | Qualitative | Y | Y | Y | Y | Y |

**Table 4. Characteristics and Analytical Coding of the Included Studies**

| Study ID | Title | Author(s), year | Country | Learning stage | Instructional components | Participation structure | Narrative-content context | Language–narrative outcomes | Socioemotional outcomes | Practice features | Learner characteristics |
| --- | --- | --- | --- | --- | --- | --- | --- | --- | --- | --- | --- |
| S1 | A randomized controlled trial to test efficacy of digital enhancements of storybooks in support of narrative comprehension and word learning | Sarı et al., 2019 | Turkey | Preschool | Digital storybooks combining narration and visual enhancements with continuous music and story-related sound effects | Receptive participation in groups of two or three; specific adult instructional support was not reported | Researcher-selected fictional stories presented through narration and visual–auditory enhancements | Music and sounds did not improve story comprehension; receptive vocabulary decreased; expressive vocabulary showed no significant change | Not measured | Moderate music–narrative integration; continuous background music may compete with narration, whereas occasional event-related sounds may be more supportive | Typically developing children aged 4–6; baseline language proficiency did not alter the multimedia effects |
| S2 | Analysis of Lyrics from Group Songwriting with Bereaved Children and Adolescents | Fiore, 2016 | United States | Primary school to adolescence | Group songwriting using participant-generated lyrics; some groups used an existing melody as a model | Collaborative and partly child-led generation in age-based groups; participants contributed orally or in writing; the facilitator provided melodic scaffolding | Bereavement narratives based on memories, shared experiences, emotions, and understandings of death | Lyric themes differed across age groups; older participants produced more diverse content and showed more mature understandings of death; no pre–post development was tested | Most participants reported enjoyment and positive feelings and believed that the songs represented their feelings and memories | High integration; lyrics directly carried grief-related memories and experiences; the one-off activity limited conclusions about change over time | Age-related differences were evident in narrative complexity; all participants had bereavement experiences; no clear age or gender differences emerged in preferences for oral or written contribution |
| S3 | Children as songwriters: The social-emotional benefits of songwriting in the elementary grades | Dweck, 2024 | United States | Primary school | Long-term songwriting curriculum involving lyrics, melody, movement, instrumentation, sharing, and performance | Collaborative and child-led generation across individual, small-group, and whole-class activities; the teacher supported melody formation and performance | Personal, emotional, family, friendship, school, and identity narratives | Songwriting supported personal storytelling, emotional expression, and the articulation of life events | Students reported greater self-confidence, empowerment, emotional expression, friendship, and classroom community | High integration; sustained, multimodal, child-authored practice with substantial content control and peer sharing | Primary-school developmental stage and prior songwriting experience; findings were based on retrospective accounts rather than formal subgroup comparisons |
| S4 | Efficacy of digital picture book enhancements grounded in multimedia learning principles: Dependent on age? | Li & Bus, 2023 | United States | Preschool | Digital picture books with auditory, visual, or combined enhancements | Receptive participation with repeated exposure; adult interaction was not reported | Researcher-assigned fictional picture books with narration and content-related auditory or visual cues | Auditory and visual enhancements improved story comprehension and book-based vocabulary; combined enhancement was less beneficial for comprehension among the youngest children | Not measured | High integration when auditory cues corresponded to story events or emotions; single enhancements were sometimes more effective than simultaneous visual and auditory enhancement | Age moderated the pattern: 3-year-olds benefited more from one enhancement at a time, whereas 4- and 5-year-olds tended to benefit more from combined enhancement |
| S5 | Embodied engagement: Fostering caring thinking in a multimodal singing-based learning environment for refugee children | Mohammadhosseini & Schmid, 2025 | Germany | Primary school | Group singing, traditional songs, rhythmic breathing, movement, clay work, shadow play, and improvised storytelling | Responsive, collaborative, and partly child-led participation in a small group; the facilitator adapted activities to children’s responses | Nonverbal, affective, cultural, and collective narratives grounded in children’s experiences and workshop themes | Sound, movement, and nonverbal storytelling supported participation despite language barriers and enabled collective narrative creation | Greater belonging, relaxation, emotional regulation, social cohesion, emotional expression, and reduced anxiety indicators were reported | High integration; sequential multimodal design, responsive facilitation, and culturally meaningful singing supported participation and connection | Refugee background, heterogeneous language experience, linguistic barriers, and needs related to anxiety, regulation, and belonging |
| S6 | Embodied narrative learning as an inclusive practice in early childhood settings: Rhythm, movement, and emotional engagement in children with autism spectrum disorder | Zacharová & Liliková, 2026 | Slovakia | Preschool | Story activities combined with rhythm, movement, musical repetition, dramatization, and spontaneous vocalization | Responsive and partly child-led participation; teachers incorporated spontaneous movement and sounds into activities; some parents were present | Educational stories supporting symbolic understanding, emotional expression, communication, and social participation | Improvements were observed in narrative understanding, symbolic awareness, story-sentence production, partial retelling, and retention | Reduced anxiety and improvements in participation, emotional regulation, safety, and social synchrony were observed | High integration; repetition, embodied entry points, and adult recognition of spontaneous behavior helped stabilize participation | ASD with substantial variation in functional speech and nonverbal communication; parental presence provided emotional security |
| S7 | Enhancing text-level reading fluency and engagement in immigrant children through a structured singing-based intervention | Choe et al., 2025 | South Korea | Primary school | Rhythmic word chanting, sentence singing, and songwriting progressing from words to sentences and narrative songs | Responsive and generative participation in a small-group program; structured modeling and graduated adult scaffolding | Reading texts and children’s personal expression organized through narrative songs | Reading fluency improved, especially accuracy, automaticity, and prosody; vocabulary, connected-sentence production, and expressive language also developed | Reading interest, attitudes, and enjoyment improved; broader socioemotional development was not directly measured | High integration; structured word–sentence–narrative progression, repeated practice, and partial child creation | Length of residence, home language, prior literacy experience, baseline Korean and reading levels, and initial reading interest may have shaped outcomes |
| S8 | Talking Together: The Effects of Traditional Māori Pedagogy on Children’s Early Literacy Development | Derby, 2023 | New Zealand | Preschool | Storytelling, shared reading, songs/chants, games, conversation, and reminiscence in a 12-week home-based literacy programme | Parent–child participation within the home, supported by structured prompts and repeated family activities. The two programme components were delivered in a crossover sequence | Culturally responsive Māori oral traditions integrating storytelling, songs, chants, family reminiscence, and bilingual interaction in English and Te Reo Māori. | Vocabulary knowledge and phonological awareness improved and were maintained at six-month follow-up; story comprehension and retelling were not reported as outcomes in this article | Not directly measured | Culturally responsive, multicomponent design; music was integrated with storytelling, conversation, games, and reminiscence, so its independent contribution could not be isolated | Eight bilingual Māori preschool children and their families. English was the dominant home language, with varying levels of Te Reo Māori use |
| S9 | “I didn’t know I have the capacity to be creative”: Children’s experiences of how creativity promoted their sense of well-being. A pilot randomised controlled study in school arts therapies | Moula, 2021 | United Kingdom | Primary school | Integrated arts therapies using music, drama, movement, visual art, storytelling, puppetry, and songwriting | Responsive, collaborative, and child-led participation within therapist-supported groups | Personal, imaginative, and emotionally difficult experiences expressed through stories, roles, songs, drawings, and puppets | Story-making and enactment supported engagement and enabled indirect expression of difficult emotions; language development was not measured | Children reported emotional expression, group bonding, empowerment, optimism, calmness, and relaxation | Multicomponent creative environment; the specific contribution of music could not be separated from other arts activities | Mild emotional and behavioral difficulties and a need for symbolic and nonverbal forms of expression |
| S10 | I Will Follow You: The Combined Use of Songwriting and Art to Promote Healing in a Child Who Has Been Traumatized | Christenbury, 2017 | United States | Primary school | Child-created drawings followed by therapist-composed responsive songs, discussion, and song revision | Responsive and child-led participation in individual therapy; the therapist validated the child’s choices and permitted changes to the songs | Personal trauma, anger, safety, self-esteem, and recovery narratives derived from the child’s drawings | Related stories and songs opened channels for verbal and creative expression; no standardized language outcome was measured | Emotional expression, self-soothing, control, self-esteem, and therapeutic connection were supported | High integration; drawing–song–discussion sequence, substantial child control, and therapist responsiveness | Trauma history, difficulty with direct verbal emotional expression, and a strong need for safety and control |
| S11 | Integration of music, yoga and massage: Investigation of their effects on preschool children’s social-emotional development and quality of life | Algül & Kılıçarslan, 2026 | Turkey | Preschool | Music activities combined with story-based yoga, breathing exercises, and massage | Responsive participation in teacher-led school groups; no generative component was reported | Educational and socioemotional stories embedded in yoga-based activities | Language–narrative outcomes were not directly measured; improvements in communication were reported indirectly | Socioemotional well-being, resilience, social competence, and psychosocial health improved, and anger-aggression decreased; some outcomes related to emotional stability, coping, anxiety, and introversion were nonsignificant | Moderate integration within a repeated multicomponent program; the independent effect of music could not be isolated | Preschool developmental stage and a general-development sample; no subgroup analysis of learner characteristics was reported |
| S12 | ‘It Is You in My Picture!’ A Holistic Approach to Six-Year-Old Children’s Art Experiences | Granö & Turunen, 2022 | Finland | Preschool | Listening to music and The Ugly Duckling, followed by collaborative drawing, short-story telling, and discussion | Receptive, responsive, and collaborative participation; small-group drawing; adults provided a safe presence and discussion prompts | Fairy-tale themes linked with children’s everyday experiences, emotions, acceptance, and peer relations | Children expressed everyday experiences through drawings, talk, and short stories; nonverbal modes supported children with weaker verbal expression | The activities supported emotional processing, belonging, acceptance, friendship, and peer relations, while also revealing existing tensions | Moderate integration; sequential music/fairy tale–drawing–story discussion, adult emotional safety, and peer co-construction | Variation in linguistic expression; some children relied more heavily on drawing, facial expression, and body language |
| S13 | Names Matter: Fostering Cultural Awareness Through Multimodal Literacy in a Predominantly White Classroom | Yang & Harper, 2026 | United States | Primary school | Repeated picture-book read-alouds combined with visual art, music, crafts, generative AI, and name-song creation | Receptive, responsive, and child-led generative participation; teacher-guided whole-class sharing | Personal and family name stories, identity, family history, and cultural awareness | Activities supported deeper text engagement, critical thinking, and narration of the meanings of children’s names; no standardized language measures were used | Cultural awareness, identity development, pride, interest, and classroom engagement were supported | High integration; identity stories were transformed into songs and multimodal artifacts with substantial child choice | Predominantly White rural Appalachian classroom; personal names, family histories, and cultural backgrounds shaped meaning-making |
| S14 | Parent-child nonverbal engagement during spoken versus sung book-sharing in preschoolers with and without autism | Liu et al., 2022 | United States | Preschool | Spoken versus sung picture-book sharing | Receptive and responsive participation in one-to-one caregiver–child dyads; caregivers served as interaction partners | Picture-book narratives presented through speech or song | Story comprehension, vocabulary, and narrative learning were not directly measured | Sung books increased visual attention in both children and caregivers; caregiver–child attention was correlated only in the sung condition for ASD dyads; movement coordination did not differ by condition | High integration; sung text directly carried book content, and caregiver participation shaped the interaction | ASD versus typical development; language-age level was associated with visual attention among children with ASD |
| S15 | Programming E-Books: Culture, English, and Scratch for Schoolchildren of Rural Taiwan | Lin et al., 2022 | China | Primary school | Scratch programming, English learning, local-culture storytelling, and creation of an animated e-book containing images, sound, music, and games | Collaborative and partly child-led generation; four groups created different story sections; university students provided instructional support | A local Siraya legend presented as an English animated picture book | English picture-book learning improved and was positively associated with Scratch performance; the specific contribution of music was not tested | Learning motivation and self-efficacy improved; broader socioemotional development was not directly measured | Multimodal, cross-disciplinary, project-based design; music was one component of the final digital product and could not be isolated | Grade 6, rural context, English as a foreign language, and baseline English and Scratch levels; no clear gender differences were found |
| S16 | Relational repair through recording: A music therapy case study with a child and parents following the October 7 attack | Hadar, 2025 | Israel | Primary school | Musical play, improvisation, layered recording, repeated listening, and family co-creation | Progression from responsive exploration to child-led generation and parent–child collaboration; the therapist provided scaffolding | Post-trauma personal and family narratives concerning safety, control, displacement, and relational repair | Symbolic meaning-making and trauma narrative processing were supported; no standardized language outcome was measured | Safety, self-efficacy, emotional openness, and parent–child relational repair were supported | High integration; cumulative recording, increasing child control, and later parental involvement supported relational processes | Displacement and trauma following collective violence, a strong need for control and safety, and parental involvement as an important contextual factor |
| S17 | The Effects of Shared Singing Picture Book Instruction on Chinese Immersion Kindergarteners’ Spoken Vocabulary Recall and Retention | Zhou & Li, 2017 | United States | Preschool | Shared singing of a Chinese picture-book story using a familiar melody; repeated teacher singing, shared singing, target-vocabulary practice, sentence strips, and enactment | Teacher-guided participation progressing from listening to shared singing and guided enactment; comparison group received shared reading | A translated and adapted version of The Little Red Hen, with target Chinese vocabulary embedded in repeated story dialogue | The shared-singing group scored higher than the shared-reading group on immediate vocabulary recall and delayed retention | Not measured | High integration; melody and repeated singing were directly aligned with story text and target vocabulary; a familiar melody was selected to limit additional task demands | 106 kindergarten children in U.S. Chinese-immersion programmes; approximately five years old and at an early stage of Chinese L2 learning |
| S18 | Poetry and Digital Media for Improving Upper Elementary African American Science Learning | Stanley, 2021 | United States | Upper elementary school | Digital storytelling, filmmaking, guided expressive reading, poetry, plays, songs, and raps embedded in a science film club | Individual and collaborative generation; students drafted, revised, storyboarded, produced, performed, and shared digital products with teacher and university-faculty scaffolding | Student-generated science stories, poems, films, songs, and raps addressing curriculum-related science topics | Performance literacy, writing, story construction, science vocabulary, oral fluency, narration, and digital storytelling/filmmaking improved | Agency and emerging literate/scientific identities were reported qualitatively; no standardized socioemotional developmental measure was used | Moderate integration within a broader multimodal design; music, songs, and raps were embedded in student-generated science narratives alongside other media | 77 fourth- and sixth-grade students in an urban parochial school; predominantly African American and from low-socioeconomic-status communitiescommunities |
| S19 | Multimodal Language Rights and Indigenous Totemic Identity: Cross-Cultural Participatory Research | Mills et al., 2025 | Australia | Preschool to primary school | Digital storytelling, singing, e-book making, dance, music-video production, drawing, and other multimodal literacy practices | Collaborative and child-generative participation; students created multimodal texts individually and in groups with teachers, Indigenous staff/Elders, researchers, and a media artist | Indigenous cultural narratives centred on totemic identity, ancestral knowledge, Country, local language, and community histories | Multimodal narrative production incorporated oral, written, visual, musical, and digital resources; no standardized developmental language change was measured | Qualitative evidence concerned cultural identity, belonging, self-determination, and relationships with Country and community; these are primarily identity- and relational affordances rather than measured developmental change | Music and singing were embedded within broader culturally situated multimodal narrative practices; strong community participation and cultural alignment were central, although music was not isolated as an independent component | Indigenous elementary students aged approximately 4.5–12 years; culturally specific Aboriginal and Torres Strait Islander school community |
